# Analysis of COVID-19 outbreak in Hubei province based on Tencent's location big data

**DOI:** 10.3389/fpubh.2023.1029385

**Published:** 2023-05-26

**Authors:** Lei Hua, Rong Ran, Tingrou Li

**Affiliations:** School of Public Policy and Administration, Chongqing University, Chongqing, China

**Keywords:** urban relation intensity, urban centrality, COVID-19, correlation analysis, Tencent's location big data, prevention

## Abstract

Rapid urbanization has gradually strengthened the spatial links between cities, which greatly aggravates the possibility of the spread of an epidemic. Traditional methods lack the early and accurate detection of epidemics. This study took the Hubei province as the study area and used Tencent's location big data to study the spread of COVID-19. Using ArcGIS as a platform, the urban relation intensity, urban centrality, overlay analysis, and correlation analysis were used to measure and analyze the population mobility data of 17 cities in Hubei province. The results showed that there was high similarity in the spatial distribution of urban relation intensity, urban centrality, and the number of infected people, all indicating the spatial distribution characteristics of “one large and two small” distributions with Wuhan as the core and Huanggang and Xiaogan as the two wings. The urban centrality of Wuhan was four times higher than that of Huanggang and Xiaogan, and the urban relation intensity of Wuhan with Huanggang and Xiaogan was also the second highest in the Hubei province. Meanwhile, in the analysis of the number of infected persons, it was found that the number of infected persons in Wuhan was approximately two times that of these two cities. Through correlation analysis of the urban relation intensity, urban centrality, and the number of infected people, it was found that there was an extremely significant positive correlation among the urban relation intensity, urban centrality, and the number of infected people, with an *R*^2^ of 0.976 and 0.938, respectively. Based on Tencent's location big data, this study conducted the epidemic spread research for “epidemic spatial risk classification and prevention and control level selection” to make up for the shortcomings in epidemic risk analysis and judgment. This could provide a reference for city managers to effectively coordinate existing resources, formulate policy, and control the epidemic.

## 1. Introduction

COVID-19 became a major issue for China in December 2019 and then turned into a global concern in the next few weeks (1, 2). In China, COVID-19 first broke out in Wuhan and then quickly spread all over China. Because the virus can survive for 14 days on the surface of the environment and objects (3), the large-scale population movement in Wuhan became the potential vectors for the spread of the virus, which led to a situation where people's lives were greatly threatened by the epidemic and severely impacted society, economy, security and so on (4–6). Existing studies also indicate that population movement is an important vector for epidemic transmission, and sudden, scattered, and large-scale human movement can transform local outbreaks of diseases into widespread epidemics (7–9). While frequent population movement facilitates the long-distance and large-scale transmission of the virus (10, 11), the movement of people between different regions plays a significant role in promoting full contact and transmission between potentially infected people and susceptible communities (12–14). Since restricting the gathering and movement of people effectively interrupted the transmission route of COVID-19, the closure of businesses, production, and even “lockdown” during the outbreak became important epidemic prevention measures. While these measures resulted in arresting the spread of the epidemic, they also seriously impacted the operation of the economy, society, and people's normal life (15). Against the background of the increasing normalization of epidemic prevention and control, it has become imperative for different regions to adopt scientific and accurate epidemic control measures customized to local conditions and times so as to balance the dual purposes of epidemic prevention and keeping the economy and society functional (16–19). Therefore, a key challenge to dealing with the outbreak has been the data and methods that should be used to study and assess the scale of the epidemic and how to understand the laws of epidemic transmission, and then choose the scientific and accurate epidemic control measures (20–23).

In recent years, infectious diseases have plagued a large number of countries and regions. At present, scholars from biology, medicine, and other fields have carried out a significant amount of research on COVID-19 focusing on the etiology, diagnosis, treatment, infectious sources, infection paths, etc. (24–26). These studies mainly consider the micro perspective of the epidemic, and the research and development cycles are quite long, which are unable to respond to the sudden and rapid characteristics of the epidemic in time (27–29). At the same time, many scholars have studied the spread of infectious diseases using predictive models. For example, Liu and Luo used the dynamic model of infectious disease to predict epidemics and proposed an early warning mechanism (30, 31). Lutz et al. predicted disease outbreaks based on influenza data from the United States, which provided a reference for decision-making in public health (32). Fan et al. predicted the spread of COVID-19 in Hubei province based on the 2013–2018 China Migrants Dynamic Survey (CMDS) (33). In recent times, with the development of big data and cloud computing technology, new methods of analyzing and predicting epidemics have emerged. Wada, Milinovich, Gluskin, and others used the Google Index to predict infectious diseases, such as influenza, dengue fever, and Ebola (16–19, 34). Liu and Chen used the Baidu index to predict the H7N9 epidemic (35–37). Guo made an application analysis of the outbreak and prediction of infectious diseases based on big data (38, 39). Hay et al. combined big data with epidemiological data to construct a risk map and built an early warning system, updating the map of the spatial distribution of infectious diseases in real-time (40).

With rapid urbanization across the world, cities are increasingly getting linked with each other. The large-scale movement of people is an important reason for the rapid spread of the epidemic, and the dense population movement between cities plays an important part in promoting the spread of the epidemic in the affected areas (41–43). Due to the changes in interactive factors such as environment, transportation mode, transmission distance, and the scale of population flow, the relationship among cities in the region is very high, which promotes the population flow to a certain extent (9, 44). Similarly, when it comes to infectious diseases, the moving population is a potential carrier of the virus, greatly increasing the possibility of the spread of the virus (45, 46). It also leads to rapid changes in the spread and scope of diseases. Existing studies have shown that epidemic research based on previous big data research methods cannot meet the needs of epidemic transmission under the current circumstances of rapid population movement. Therefore, within epidemic transmission prediction studies, it has become a research focus to use big data along with geographical location to analyze epidemic transmission (47, 48). Based on big data, mining population travel patterns can reveal how infectious diseases will spread between regions, which can aid in containing the spread. Furthermore, these data analyses can provide a reference for city managers to implement scientific and accurate epidemic control measures before catastrophic epidemics break out or reoccur.

Research on the prediction and transmission of infectious diseases has therefore become a key subject of interest the world over. Existing research often relies on epidemic surveillance data and statistical reports, but seldom do they approach the problem from the perspective of geography, and big data with location attributes are rarely used (49). Previous studies have shown that big data from mobile phones is one of the best information sources for large-scale population movements (50, 51), and using big data to study COVID-19 is one of the most convenient methods (52–54). In this study, we examined the prevalence of COVID-19 in Hubei province from 20 January to 16 March 2020, by taking Hubei province as the research area and measuring the population flow by urban relation intensity and urban centrality provided by Tencent's location big data. This study analyzed the spatial distribution characteristics and correlation between the intensity of urban relations, urban centrality, and the number of infected people in Wuhan and other cities in Hubei province, and also explored the relationship between urban relation intensity, urban centrality, and regional epidemic transmission. By providing new insights into the spread of the epidemic and its prevention, this study provides a reference for city managers to understand the dynamics of epidemic transmission to adopt scientific and accurate epidemic control measures.

## 2. Materials and methods

### 2.1. General situation of the study area

**Figure 1:**
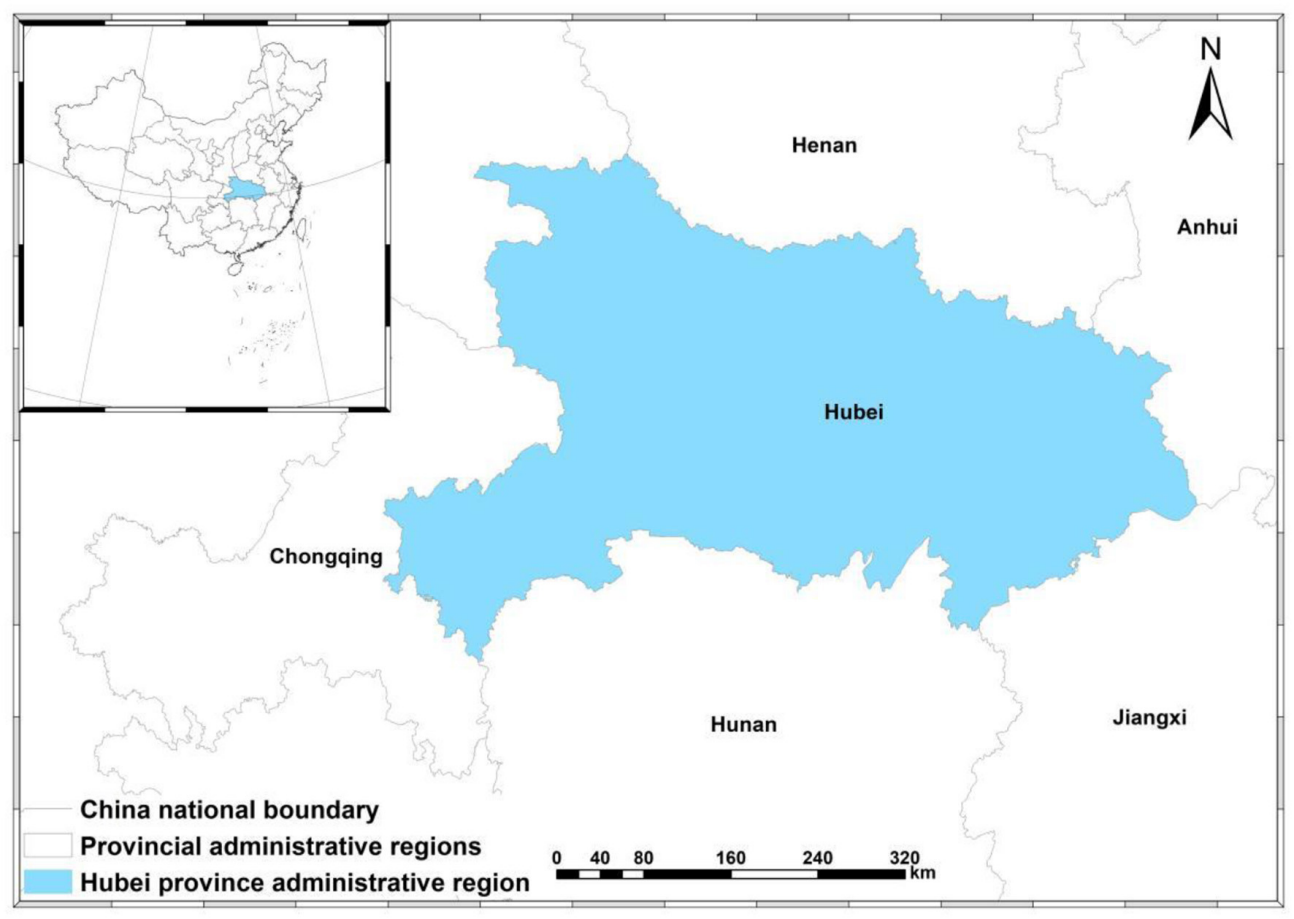
Location of the study area, Hubei province, China.

As shown in Figure 1, Hubei province is located in central China with beautiful scenery and a comfortable climate (55, 56), bordering Anhui province in the east, Chongqing in the west, Shaanxi province in the northwest, Jiangxi province and Hunan province in the south, and Henan province in the north. It lies between 29°01′53″-33°6′47″N and 108°21′42″-116°07′50″E. It is ~740 km long from east to west and 470 km wide from north to south with an area of 185,900 sq. km accounting for 1.94% of the total area in China.

### 2.2. Data

#### 2.2.1. Tencent's location big data

Tencent's location big data (https://heat.qq.com/qianxi.php) is a new population mobility research platform with 990 million monthly active users, providing location information derived from the Internet+ and cloud computing (57, 58). The generated data can be used to analyze the geographical location changes, i.e., population mobility. Key indicators such as the direction, number, and mode of mobility can be obtained through terminal analysis. Tencent's location big data has, therefore, become the flagship of population mobility research. The dynamic nature of the data enables researchers to intuitively analyze the population mobility, urban centrality, and urban relation intensity in the cities in the research region. The data for this study was mainly obtained by crawling the latitude and longitude of the sample cities by code input, and the crawled data was cleaned, merged, and deduplicated.

As the COVID-19 epidemic spanned across festival and holiday periods, including working days, the use of population mobility data during those periods has an inevitable impact on the research results. Therefore, the population movement through various means of transportation in Hubei province from 1 to 14 April 2017 from Tencent's location big data was selected for analysis. The data samples included festivals, holidays, and working days, which could then fully reflect the characteristics of population mobility.

#### 2.2.2. COVID-19 infection data

In this study, the number of infected people in Wuhan was sourced from the daily announcements made by the Hubei Provincial Health Committee from 20 January to 13 March 2020. Hubei Provincial Health Committee Map (http://wjw.hubei.gov.cn/bmdt/ztzl/fkxxgzbdgrfyyq/xxfb/) was also referred.

### 2.3. Methods

#### 2.3.1. Urban relation intensity

As the direct manifestation of the interconnection and interaction between cities, population migration has been a hot issue in the field of urban networks. The urban relation intensity can be denoted as the relation between the two analysis units. The stronger the relation intensity, the greater the spatial interaction intensity, and the stronger the interdependence between the two cities. Specifically, it is manifested as the average of the population flow data and the sum among the cities. Since the main carrier of the epidemic spread is population movement, this paper attempted to investigate the relationship between the strength of urban relation intensity calculated by population movement data and the spread of the epidemic (59–61) using the formula:


(1) $$\begin{array}{l} S_{ab}=\left[S_{a-b}+S_{b-a}\right]/n \end{array}$$

where *S*_*a*-*b*_ is the number of people moving from city *a* to city *b*, *S*_*b*-*a*_ is the number of people moving from city *b* to city *a*, and *n* is the number of days.

#### 2.3.2. Urban centrality

In recent years, urban network research has become a new paradigm in urban studies. Scholars have focused on the importance and characteristic attributes of urban nodes and have constructed urban centrality to analyze the hierarchical structure and the relevance of urban networks. Urban centrality refers to the connection dominance of node cities in the regional network. Population mobility is used to show the centrality of node cities, which includes the mobility and aggregation capacity of a city in the regional network. It is the sum of the connection strength of urban points in the urban network, and the centrality can reflect the level and status of nodes cities in the network. In this context, urban centrality is a unified representation of resource aggregation and diffusion in the urban network. The higher the urban centrality, the higher its status in the urban network, and the more possible its population, capital, information, and other resource elements will aggregate to the city with the highest urban centrality. Similarly, the relevant resource elements will also diffuse from there, which is also the manifestation of urban centrality. Therefore, this study mainly focused on the spread of the epidemic in the urban circle network of Hubei province, trying to discover the spatial connection between urban centrality and the spread of the epidemic in the urban network through this indicator (62–64) using the formula:


(2) $$\begin{array}{l} S_{\alpha }=\sum_{i=1}^{n}\left(S_{\alpha i}+S_{i\alpha }\right)\left(i=1,2,3\cdots n,n\neq i\right) \end{array}$$

where *Sa* is the sum of urban relationship intensity between city *a* and city *i*, *Sai* is the sum of urban relationship intensity between city *a* and other cities, and *Sia* is the sum of urban relationship intensity between city *i* and other cities.

#### 2.3.3. Natural breaks

Natural Breaks is a classification method based on the inherent natural break points in data. It classifies the data according to the similarity between the values and combines similar values most appropriately. It maximizes the difference between groups by calculating the variance of each class by setting the boundary at the place where the difference in data value is relatively large and dividing the elements into several classes (65, 66).

#### 2.3.4. Correlation analysis

It is often necessary to analyze the correlation between two or more variables in sociology, statistics, etc. If either one or both variables are classified variables, then the Spearman method needs to be used. If both variables are continuous variables, then the Pearson analysis is more suitable. Since the data used in this study have continuous variables, the Pearson method was adopted for correlation analysis, where *P* < 0.05 was considered statistically significant (67, 68).

#### 2.3.5. Research framework

**Figure 2:**
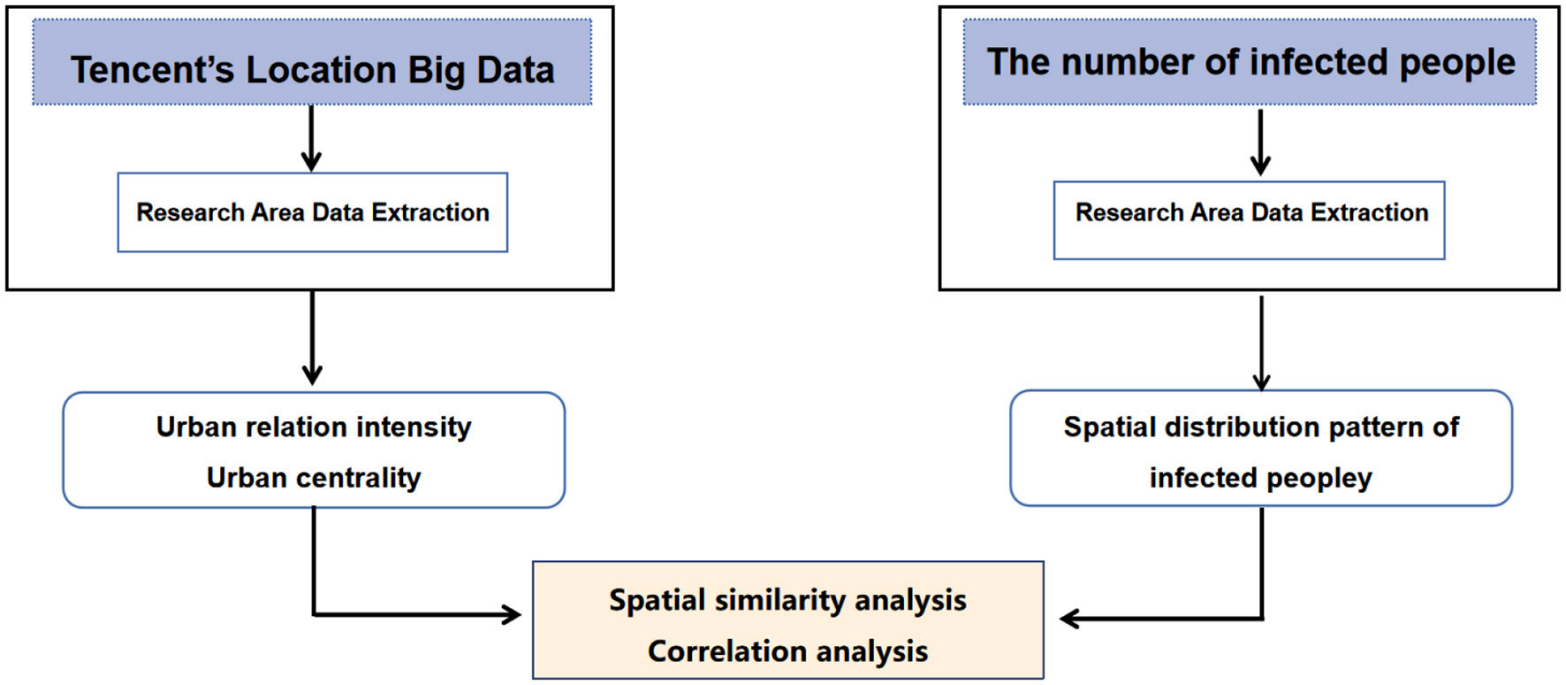
Research framework.

The research framework of this study is illustrated in Figure 2.

## 3. Results

### 3.1. The spatial pattern of urban relations intensity in Hubei province

**Figure 3:**
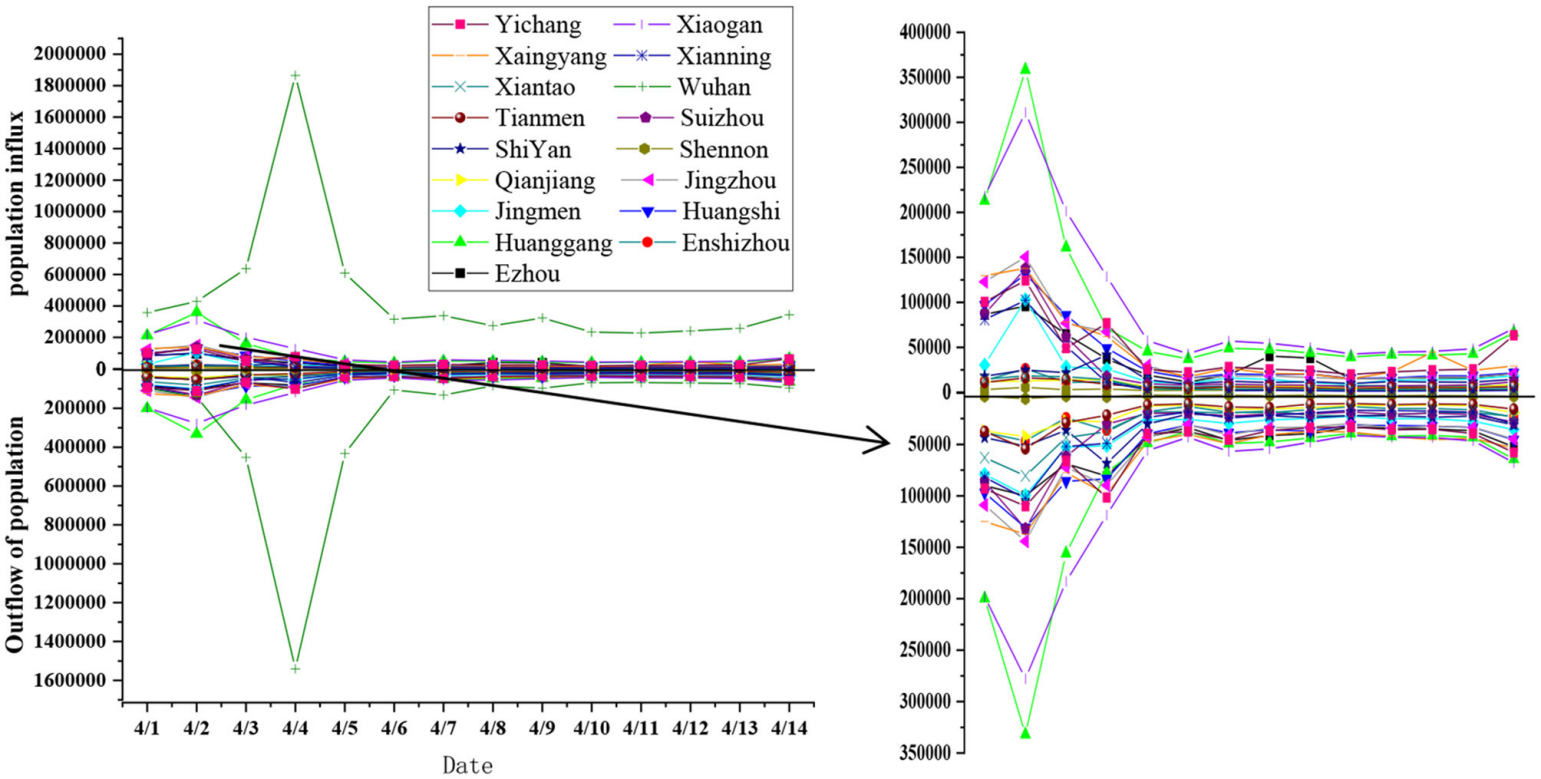
Population mobility in urban areas and suburbs of Hubei province.

The overall spatial distribution showed that population mobility was mostly concentrated in eastern Hubei province, especially in Wuhan, Xiaogan, Huanggang, and other cities located in this region. Population mobility appeared to be relatively stable and balanced, and there was no one-way flow in or out of each city. However, between cities, due to the lack of resource availability and economic development, there was a significant imbalance in population mobility. Wuhan experienced extreme flow, which was multiple times higher than that of other cities. As seen in Figure 3 and Table 1, Shennongjia, Tianmen, and Qianjiang accounted for 0.31, 1.36, and 1.43% of the total population mobility, respectively. Central Hubei had a moderate proportion, e.g., Jingzhou and Jingmen accounted for 5.11 and 3.25% of the total population flow, respectively. The proportion of population flow in eastern Hubei province was the highest. The total population mobility of Wuhan, the provincial capital, was 36.92%, which was 7 times higher than that of Xiangyang and Yichang, the two vice-provincial cities. Therefore, the advantage of the central position was obvious. Population mobility of Wuhan and Huanggang accounted for nearly half of the total population mobility.

**Table 1:** Data table of population flow in Hubei Province based on Tencent's location big data.

**City**	**Population influx**	**Outflow of population**	**Total flow of population**	**As a percentage of total**
Shennongjia	43,518	40,252	83,770	0.31%
Tianmen	103,448	262,256	365,704	1.36%
Qianjiang	110,743	272,323	383,066	1.43%
Enshi	131,222	345,847	477,069	1.78%
Xiantao	100,306	393,112	493,418	1.84%
Shiyan	223,429	439,406	662,835	2.47%
Xianning	263,041	493,206	756,247	2.82%
Jingmen	320,976	550,974	871,950	3.25%
Suizhou	387,581	520,134	907,715	3.38%
Ezhou	496,748	734,722	1,231,470	4.59%
Huangshi	552,809	753,760	1,306,569	4.87%
Jingzhou	600,967	770,940	1,371,907	5.11%
Yichang	632,675	765,566	1,398,241	5.21%
Xiangyang	656,864	876,110	1,532,974	5.71%
Huanggang	1,259,471	1,220,436	2,479,907	9.24%
Xiaogan	1,373,668	1,277,689	2,651,357	9.88%
Wuhan	6,451,572	3,455,877	9,907,449	36.92%
Total	13,665,520	13,172,610	26,838,130	100.00%

According to Figure 3 and Table 1, the cities with the highest flow were Wuhan, the provincial capital located at the regional core of Hubei province, and its neighboring cities of Xiaogan and Huanggang. Their total population mobility accounted for 56.04% of the total population flow. While secondary floating cities were mainly located in southeastern and central Hubei province, second-and third-tier cities and sub-provincial cities were close to the core cities, including Xiangyang, Yichang, Jingzhou, Huangshi, and Ezhou. Their average population flow accounted for only 5% of the total population flow. The cities with the lowest population mobility were Shennongjia, Tianmen, Qianjiang, Enshi, and Xiantao. These cities, located in western and central Hubei province are far away from the core cities and characterized by low population density and an underdeveloped economy. The population mobility of these cities was usually outflow of population, and there was an increasing trend in the number of people entering the cities only on holidays.

The urban relation intensity indicates the degree of development and interaction among cities in a region. In this study, we utilized ArcGIS 10.2 to inspect the population mobility data of 17 prefectures in the Hubei province. By using the natural breakpoint method, the urban relation intensity of Hubei province was classified into five levels, while the urban relation intensity in the spatial network showed a three-dimensional trend. The relation intensity degree of urban nodes was characterized as “decreasing from east to west”. The regional space showed the central radiation pattern with Wuhan at the core, and the region took the form of a single-center integration.

**Figure 4:**
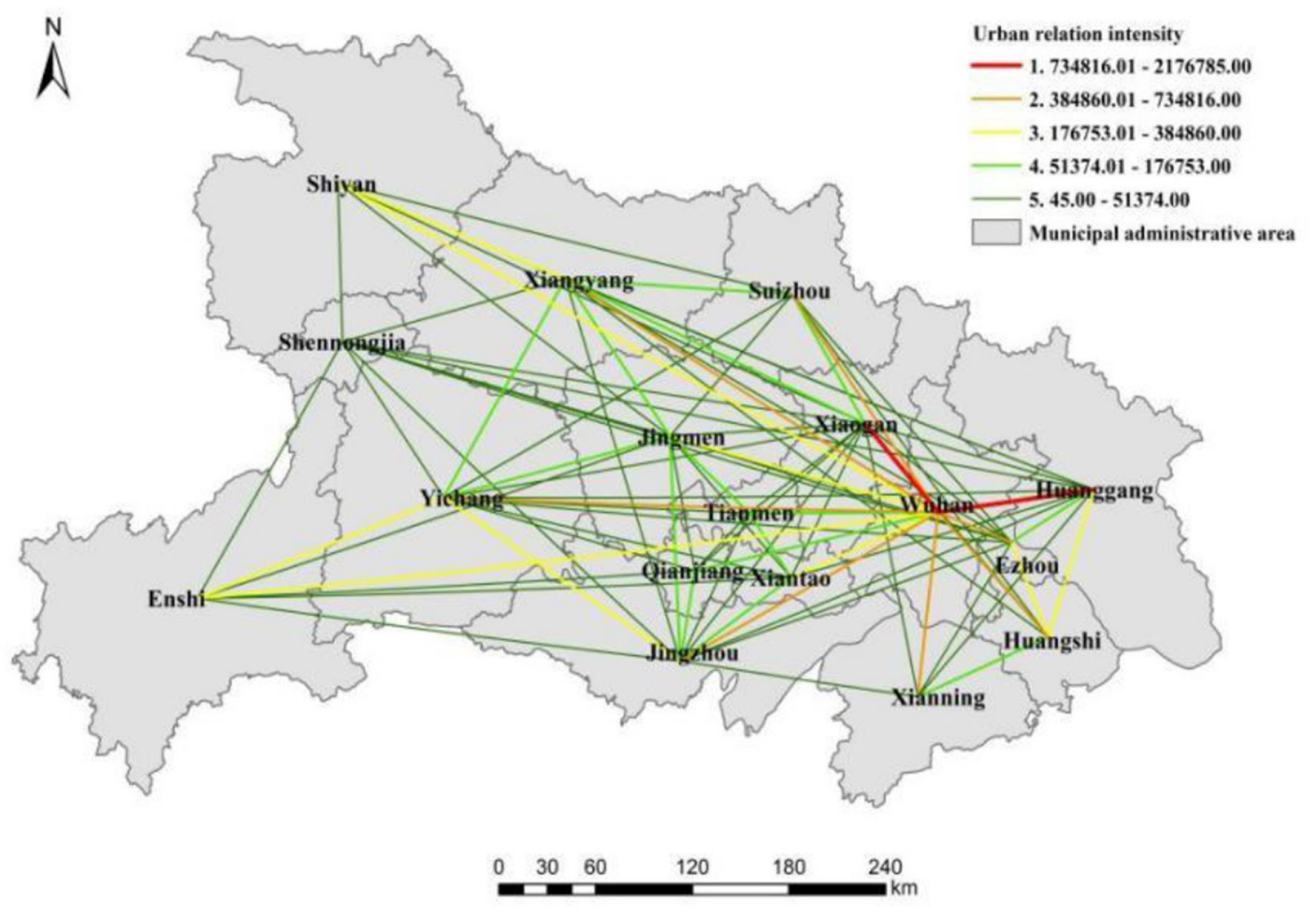
The spatial pattern of urban relation intensity in Hubei province.

As seen in Figure 4, the spatial pattern of urban relation intensity in Hubei province took Wuhan as the center, and revealed a radiated connection axis distribution embodying the spatial relation characteristics of an “axis and spoke”. It formed a point-to-point spatial network structure and the whole spatial pattern presented a circular connection. The spatial relation pattern level was particularly obvious at this point with a “siphon effect”. The level 1 and level 2 axes with higher urban relation intensity were the relation axes of 10 cities of Wuhan, except Shiyan, Qianjiang, Jingmen, Tianmen, Shennongjia, Enshi, and Xiantao, which indicated that Wuhan was at the core of regional space.

Except for Wuhan, the urban relation intensity of 16 other cities was relatively weak and most of them were at level 5; especially in Shennongjia and 16 other cities in Hubei province where the urban relation intensity was at level 5. In the spatial pattern of urban relation intensity, Xiangyang and Yichang showed the characteristics of high urban relation intensity with cities in eastern Hubei province and low urban relation intensity with cities in western Hubei province. Although it is located in the border area of western and central Hubei province, the urban relation intensity with western Hubei province was mostly at level 5, and only the urban relation intensity between Yichang and Enshi reached level 3. In particular, Shiyan, Shennongjia, and Enshi in western Hubei province had the lowest relation intensity with other cities; only the intensity of Enshi with Wuhan and Yichang, and the intensity of Shiyan with Wuhan reached level 3, and the relation intensities with other cities were all at level 5. In the spatial analysis of urban relation intensity in Hubei province, the extent of the relationship between cities in central Hubei province and other prefectures was relatively average, and the urban relation intensity between cities was mostly at level 4. For example, the urban relation intensity between Jingmen and Yichang, Jingzhou, Tianmen, and Xiangyang was at level 4. Since the geographical location of eastern Hubei is close to the regional core city of Wuhan, its location advantage was very obvious, and the commuting cost with Wuhan was slightly lower than that of the central and western cities in Hubei province. Therefore, the urban relation intensity was higher than that of other areas, and the urban relation intensity was beyond level 3.

As seen in Figure 4, there was an obvious difference in urban relation intensity between Wuhan and other cities, indicating that the basic network agglomeration was initially formed in Hubei province. The spatial pattern of urban relation intensity in the cities revealed the unipolarity of cities with Wuhan as the center, the imbalance in the development of urban agglomeration, and the development of the Hubei metropolitan area in a single core stage. There was still a big gap between the spatial patterns of the multi-level urban network connection system. The whole spatial pattern, except Wuhan, had faults, and the urban relation intensity between Wuhan and the other cities showed a decreasing trend from east to west. The urban relation axes between Wuhan and Huanggang, and the urban relation axes of Wuhan and Xiaogan, were more prominent, and the urban relation intensity was at level 1. Comparatively, the urban relation intensity between Wuhan and Xiaogan was the highest, which was several times, even dozens of times, more than that of other cities. The urban relation intensity between Wuhan and Huangshi, Xianning, Ezhou, Jingzhou, Yichang, Xiangyang, and Suizhou was at level 2. The cities with level 3 urban relation intensity were Shiyan, Enshi, Xiantao, and Jingmen, whereas Qianjiang and Tianmen were at level 4. Only the intensity between Shennongjia and Wuhan was at level 5, which was the lowest in the whole Hubei province.

### 3.2. Spatial pattern of urban centrality in Hubei

**Figure 5:**
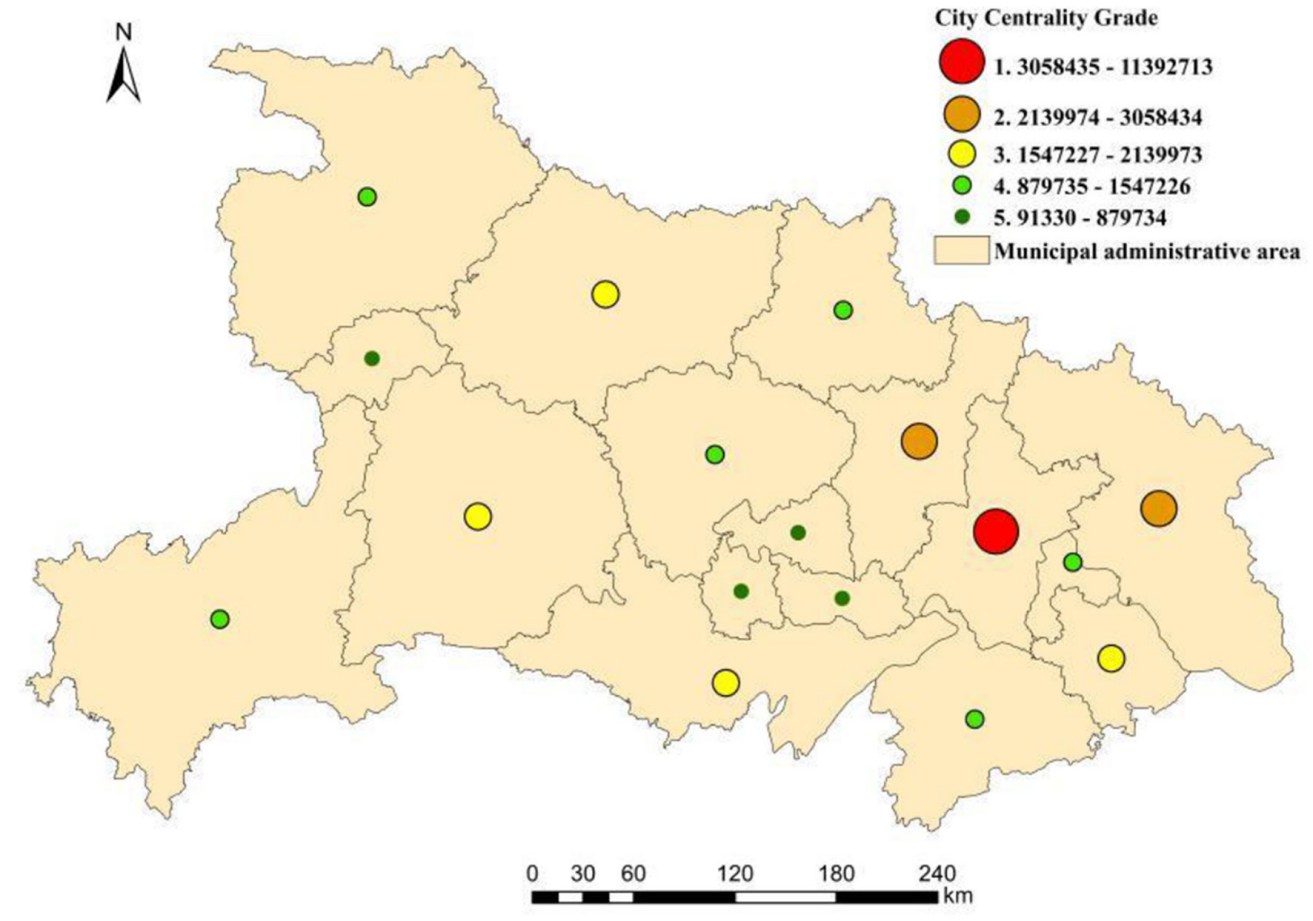
Urban centrality of Hubei province.

The urban centrality represents the inter-city radiation and relation ability based on population mobility. The higher the centrality is, the stronger the spatial radiation function of the urban point. On the contrary, the larger the radiation range is, the lower the urban point centrality and spatial radiation function. Using the ArcGIS software to measure and analyze the urban centrality of Hubei province, and using the natural breakpoint method, the urban centrality of 17 cities in Hubei province was divided into 5 levels. The specific level of urban centrality is shown in Figure 5.

There was a negative correlation between the level of urban centrality and the number of urban points in Hubei province. The higher the level of urban centrality is, the less the number of urban points and the level of urban centrality is significant. In the network, the urban centrality presented a single-center network pattern of “one big and two small”. Wuhan occupied the highest level in the spatial pattern of regional centrality, which demonstrated that it was the core city of the regional spatial pattern of Hubei with the largest spatial radiation capacity and range. Further, the adjacent cities of Wuhan were in the core position in the spatial relations of the whole region. They had a dominant position in the network and could lead the strengthening of regional spatial relations and the development of regional integration. The city centers of Huanggang and Xiaogan were at level 2. The two cities, both located in eastern Hubei province, were geographically close to Wuhan. The urban centrality of Wuhan was approximately four times of these two cities. The cities at level 3 urban centrality mainly included Xiangyang, Yichang, and Jingzhou in central Hubei and Huangshi in southeastern Hubei province. The urban centrality value of Wuhan was about 5.7 times the average of these four cities.

The cities with a low level of urban centrality were mainly located in western and central Hubei province. For example, the cities at level 4 urban centrality (e.g., Shiyan, Jingmen, Ezhou, Xianning, Suizho, and Enshi) accounted for 35% of the total number of cities. There was a positive correlation between the change in urban centrality and the change in geospatial distance from Wuhan. The further the distance was, the lower the urban centrality was. It indicated that urban centrality decreased as distance increased. It was evident that geographical location played an important role in the composition and structure of the urban network. The core cities were of utmost importance to the formation and organization of the regional network and reflected the “spatial spillover” effect of the core city. The cities at level 5 were Enshi, Xiantao, Tianmen, and Qianjiang. On the other hand, Wuhan was ~20 times the average value of these four cities which either had a small population or poor economic development. It indicated that the level of urban centrality was closely related to population and economic welfare.

### 3.3. The spatial pattern of the infected people in Hubei province

With the official nnouncement of COVID-19 in Wuhan in December 2019, it became a matter of national concern in China. The virus first spread to Wuhan and then all over China and the whole world. Consequently, people's lives and safety, the global economy, and public security were greatly threatened. Since the outbreak happened first in Wuhan, Hubei province, the situation there was the most severe in the world. As of 16 March 2020, there were 67,799 infected patients, and 3,111 had died globally.

**Figure 6:**
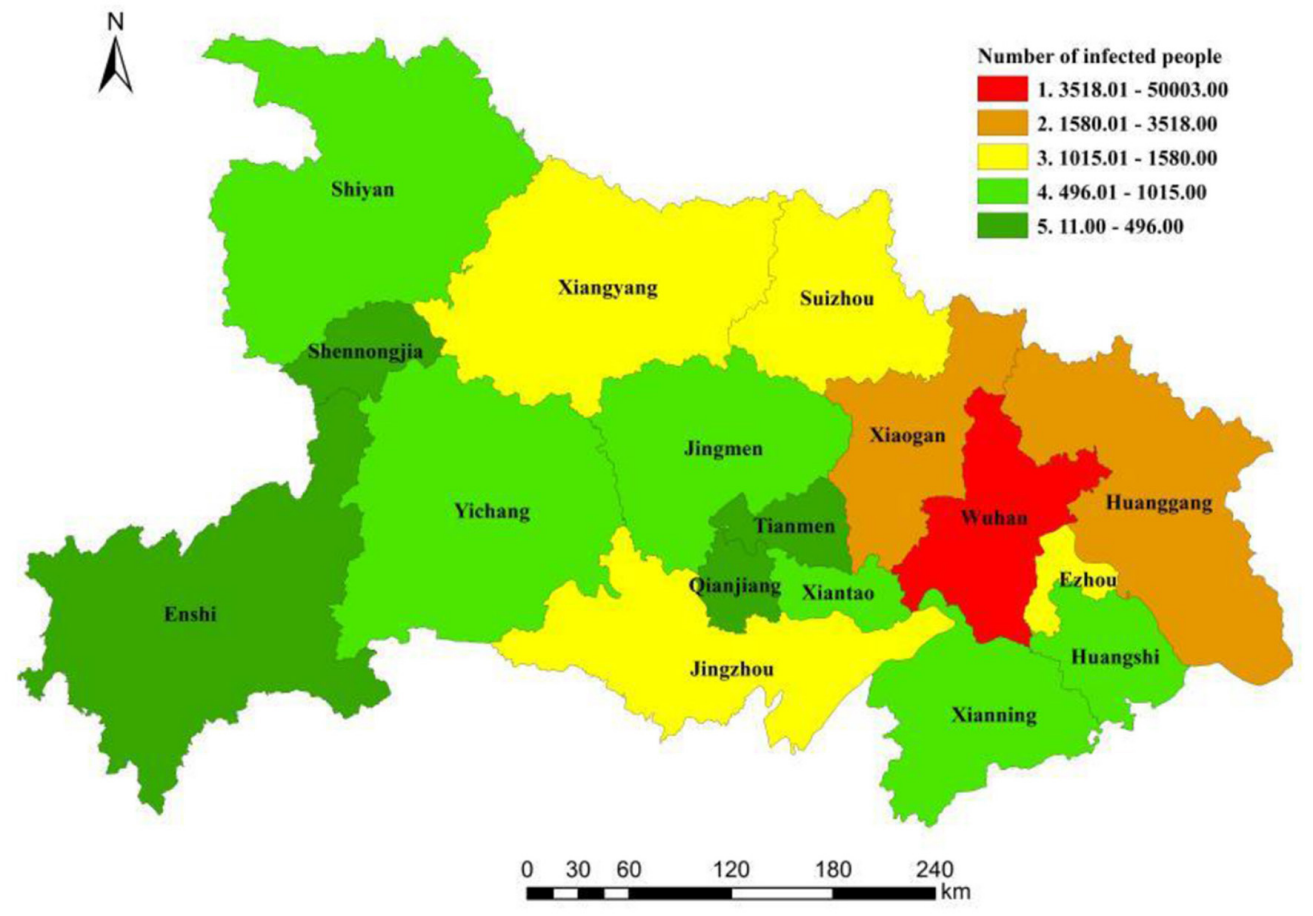
Spatial distribution of the infected people in Hubei province from 20 January to 16 March 2020.

The number of infected people in Hubei province was analyzed by using ArcGIS software. The areas were categorized under 5 levels using the natural breakpoint method, as shown in Figure 6.

Since the virus did not show considerable symptoms during the incubation period, it has gone unnoticed in Wuhan. And due to the inter-city links, the epidemic rapidly spread throughout the Hubei province. The number of infected people in the remaining 16 prefectures and states of Hubei province increased each day and showed an exponential growth trend after 31 January 2020. The largest increase was recorded in Xiaogan and Huanggang located in eastern Hubei province, followed by Suizhou, Xiangyang, and Jingzhou in central Hubei. The lowest growth rate was in Shennongjia in western Hubei province due to its low population density and underdeveloped economy.

Figure 6 shows the spatial distribution of the number of infected people in Hubei province. The numbers were higher in the east and lower in the central and western regions. There was a hierarchical structure and a single-center spatial pattern of “one big and two small” in regional infection was evident. On the contrary, the number of cities with a higher amount of infected people was quite low. Moreover, Wuhan, as the core city of the region, had the highest level of infection with 50,003 infected people. The number of infected people in Xiaogan and Huanggang, located in eastern Hubei and graded as level 4, were 3,518 and 2,907, respectively. The numbers were twice as many as those in level 3 of Xiangyang, Suizhou, and Jingzhou in the central Hubei province. The infection level in western Hubei province was generally low (level 4 and level 5), e.g., Shiyan and Enshi in the northwest and southwest Hubei. Shennongjia, located in western Hubei, had the least number of infected people, which was only 11.

### 3.4. The spatial pattern similarity and correlation analysis

#### 3.4.1. The spatial similarity analysis

Based on the above analysis, it was evident that there was a high similarity in the spatial pattern of urban relation intensity, urban centrality, and the number of infected people. The overall spatial pattern was consistent with the structure, and the three elements had the same spatial distribution characteristics. Hubei province showed the spatial distribution characteristics of Wuhan as the core, whereas Huanggang and Xiaogan were the two wings, denoted as “one big and two small”. The spatial distribution decreased from east to west, with the highest numbers in the northeast and the lowest in western Hubei province. Comparing urban relation intensity between Wuhan and the other cities, it was evident that with the expansion of the epidemic, the number of infected people was consistent with the characteristics of the urban relation intensity between the cities. For example, Wuhan had the highest level of relation with Xiaogan and Huanggang, and the number of infected people was also the highest. Furthermore, Qianjiang and Tianmen in central Hubei had the lowest relation intensity with Wuhan which was only at level 4, and the number of infected people was therefore correspondingly small. Similarly, Shennongjia had the lowest relation with Wuhan, and the number of infected people was also the least. Therefore, from the comparison of the urban centrality and the number of infected people in Hubei province, the number of infected people was highly consistent with the level of urban centrality. The urban centrality of Wuhan was the highest, and the number of infected people was also the highest. Huanggang and Xiaogan were second in terms of spatial levels, and so were their infection levels. The urban centrality of Shennongjia, Tianmen, and Qianjiang was relatively low, and they were all at level 5 in the network level of urban centrality. Therefore, these cities were also at level 5 in the spatial level of the number of infected people in Hubei province.

#### 3.4.2. Correlation analysis

The correlation analysis revealed that there was a high correlation between urban relation intensity, urban centrality, and the number of infected people, as seen in Table 2.

**Table 2:** Correlation analysis between the number of infected people, urban relation intensity, and urban centrality of the cities in Hubei province.

**Correlation analysis**	**Urban relation intensity**		**Urban centrality**	
	*R* ^2^	***P***	*R* ^2^	***P***
Infected people	0.976	0.000	0.938	0.000

In Table 2, *R*^2^ is the correlation coefficient, and its range is [−1, 1]. There was an absolute negative correlation if *R*^2^ = −1; there was a negative correlation if −1 < *R*^2^ < 0; there was an absolute non-correlation if *R*^2^ = 0; a correlation for 0 < *R*^2^ < 1; there was an absolute positive correlation if *R*^2^ = 1. As seen in Table 2, the closer *R*^2^ was to 1, the higher the degree of correlation was. *P* indicates the significance, where *P* < 0.001 meant it was extremely significant, 0.001 < *P* < 0.01 meant very significant, 0.01 < *P* < 0.05 indicated general significance, and *P* > 0.05 meant there was no significance.

According to the correlation analysis of urban relation intensity between Wuhan and the other cities in Hubei province, considering the number of people infected by COVID-19, it was found that the urban relation intensity was an independent variable, while the number of infected people was a dependent one. The *R*^2^ value was 0.976, which revealed that the urban relation intensity between Wuhan and the other cities had a positive correlation with the number of infections. The *P*-value was smaller than 0.01, which indicated that there was an extremely significant positive correlation between the urban relation intensity and the number of infections. For the correlation analysis between urban centrality and the number of infected people, urban centrality was taken as the independent variable while the number of infected people was the dependent variable, where *R*^2^ was 0.938. It also demonstrated that there was a positive correlation between urban centrality and the number of infected people. Similarly, where the *P*-value was <0.01, the intensity of urban linkage had an extremely significant positive correlation with the number of infections. As a result, there was an extremely significant positive correlation between the urban relation intensity, the level of urban centrality, and the number of infected people. The correlation between the strength of urban relation intensity and urban centrality with the spread of epidemics was demonstrated. This finding can provide a new perspective for early control and prevention of infectious diseases.

## 4. Discussion

### 4.1. Analysis of the relationship between city connectivity and the spread of the epidemic

From the perspective of spatial distribution, the spatial pattern of the number of infected people was very similar and highly correlated with the strength of urban relations and urban centrality. The overall spatial pattern was consistent with the structure, and the three elements had the same spatial distribution characteristics. It mainly presented a spatial distribution pattern of “one big and two small” with Wuhan as its core and Huanggang and Xiaogan as the two wings. That is, the spatial distribution of the number of infected people mainly spread outward from Wuhan at the center, decreasing from east to west. The northeast regions suffered the most while the western regions suffered the least. This was because the development of the Wuhan metropolitan area promoted the opening of intercity railways, which reduced the intercity commuting cost, thereby increasing the volume and frequency of population movement from cities adjoining Wuhan to Wuhan (9, 69). As a center of the regional economy, Wuhan's high population density and rapid population movement resulted in the “siphon effect” that served as a catalyst for the spread of infectious diseases (70, 71), On the other hand, for the cities in the northwest, such as Shennongjia and Enshi, due to the long straight-line distance and high commuting cost, the urban centrality and urban connection intensity levels were reflected in the scale of epidemic infection in the region. This also proved that the scale of epidemic infection was highly correlated with urban relation intensity and urban centrality (72–74).

Existing research also suggests that accessibility is responsible for the rapid spread of the epidemic. In addition, it is not easy to isolate infected people for testing because of the incubation period, which was one of the main reasons for the implementation of the “lockdown” policy in many cities (75). Given that the traditional methods of epidemic detection and control have been unable to check the spread of COVID-19 (76), it is imperative to identify the scale and transmission pace of the epidemic based on new data and methods, quickly and promptly isolate the hardest-hit areas and secure the areas that are at high risk of infection spread (72, 77). At the same time, it is also essential to monitor areas closely connected with the hardest-hit cities to prevent the spread of the epidemic on a larger scale (78). In China, urban development plans need to pay attention to the potential impact of epidemics on cities even while strengthening urban connectivity and linkages. City planners must make full use of novel technologies and methods such as big data to provide a reference for city managers to understand the law of epidemic transmission and adopt appropriate scientific and accurate epidemic control measures (79–81).

### 4.2. Prevention and control of epidemic outbreak transmission using big data technology

When a new infectious disease epidemic emerges, it generally takes a long time for specific vaccines and relevant treatments to take effect. During this time, the most direct and effective emergency prevention and control measures would still be based on isolation, prevention, and control strategies that restrict population movement (82, 83). However, the negative impact of large-scale restrictions on urban production, life, and economy cannot be ignored (84). Therefore, it is urgent to grasp the temporal and spatial law of epidemic transmission quickly and effectively to implement hierarchical prevention and control according to the spatial differences of epidemic risks. In addition, based on the level of the epidemic and the transmission pace, it is important to quickly determine the infectious strength and transmission pace of the epidemic. The compulsory control and regulation of social production and life locally will ensure that people remain in a relatively scattered, fixed, and static state, thus preventing chaos and disrupting the epidemic transmission chain in a complete, rapid, and cohesive manner (85, 86). The management department of Hubei province has mainly focused on Wuhan since the early spread of the epidemic and has not paid enough attention to the situation in other cities closely related to Wuhan (73, 87). Therefore, they have not formulated comprehensive, reasonable, and differentiated epidemic prevention and control policies. In the later stages of the epidemic, the outbreak in other cities in Hubei province led to a shortage of medical personnel and equipment, adversely affecting the control of the epidemic because of the misjudgment and insufficient preparation of the government departments (88). Therefore, under strict privacy protection and confidentiality protocols, it is worth exploring the full use of spatial big data methods such as mobile phone signaling for future research and crisis management (89). Precise prevention and control measures should be put in place making use of spatial risk classification of epidemic outbreak transmission to identify and monitor high-risk areas (90), and reduce the impact of the spread of the epidemic on socio-economic life (91).

### 4.3. Limitation

For major public health crises such as COVID-19, emergency management and control often require multidisciplinary research that includes various disciplines such as pathology, epidemiology, geographic information science, psychology, and behavioral science (86). Combining epidemiology with geographic information technology, this study made preliminary and useful attempts to examine the nature and pace of the epidemic. Therefore, it is a must for epidemic prevention and control strategies. There is an express need to have a coordinated, classified, and collaborative system that provides a scientific and technological guarantee using spatial big data. This can significantly contribute to China's success in fighting COVID-19 (92, 93). In addition, China's prevention and control strategies and local practices toward COVID-19 have provided an effective reference for other countries. Moreover, with the extension of Internet+, more and more disciplines can be used to study these hot issues by making full use of their advantages. While big data is useful for the early prediction and detection of epidemics, the scale of infection caused by different disease factors is also different (55, 94, 95). Due to the limitation of data accessibility, we selected the population movement data of 2017, and future studies should select data closer to the outbreak or in real time for the study, which can improve the accuracy of the study. Future research should also simultaneously use multi-source fusion technology, and fully consider the interference of multiple factors such as environment, transportation, and economy, in the study of the spread and development of the epidemic using real-time population movement data and infection coefficients (96).

## 5. Conclusions

Based on Tencent's location big data, this study researched the spread of the epidemic with the goal of “spatial risk classification and the selection of prevention and control”, focusing on the number of infected people as an important indicator to assess the epidemic situation. This study introduced accurate data about the number of infected people in 17 cities in Hubei province and presented the spatial distribution of epidemic transmission, but also calculated the urban connection and centrality between cities at all levels and Wuhan based on Tencent's location big data and analyses the correlation with the number of infected people to make up for the shortcomings in epidemic risk analysis and judgment. The findings of this study would greatly benefit the effective coordination of available resources, policy development, and the management and control of any epidemic. The key takeaways are as follows:

1. The spatial distribution of infected people, the spatial distribution of urban connection, and centrality taken together present the spatial distribution characteristics. Specifically, all of them show the spatial distribution of “one big and two small” with Wuhan at the core and Huanggang and Xiaogan as its two wings. Their values reveal the spatial distribution features decreasing from east to west with the northeast region being the highest and the west being the lowest.
2. Studies on the correlation between urban relation intensity, urban centrality, and the number of infected people indicate that there is an extremely obvious positive correlation between the urban relation intensity, urban centrality, and the number of infected people. And the correlation coefficients were 0.938 and 0.976, respectively. It demonstrated that the calculation of urban relation intensity and urban centrality based on Tencent's location big data was able to predict the pace and scale of epidemic transmission, which provides a new perspective on the early detection and prevention of infectious diseases.

This research not only verified the correlation between the spatial distribution of population movements and the number of epidemic infections based on big data but also analyzed the epidemic transmission pace in Hubei province. The results indicate that it is very important for cities to start from their urban relation intensity with the outbreak area and neighboring cities at different stages of the spread of the epidemic and conduct real-time research and judgment of the spatiotemporal pattern about the spread of the epidemic based on big data with geographical attributes. Only such systematic research can help formulate scientific and effective epidemic prevention and control and enable decision-making based on big data for future crisis management.

## Data availability statement

The raw data supporting the conclusions of this article will be made available by the authors, without undue reservation.

## Author contributions

RR: conceptualization and writing—review and editing. LH: methodology and formal analysis, data curation, and writing—original draft preparation. TL: project administration.
